# Hand proximity facilitates spatial discrimination of auditory tones

**DOI:** 10.3389/fpsyg.2014.00527

**Published:** 2014-06-11

**Authors:** Philip Tseng, Jiaxin Yu, Ovid J. L. Tzeng, Daisy L. Hung, Chi-Hung Juan

**Affiliations:** ^1^Institute of Cognitive Neuroscience, National Central UniversityJhongli City, Taiwan; ^2^Taipei Medical University-Shuang Ho Hospital, Brain and Consciousness Research CenterNew Taipei City, Taiwan; ^3^Institute of Neuroscience, National Yang Ming UniversityTaipei, Taiwan; ^4^Institute of Linguistics, Academia SinicaTaipei, Taiwan

**Keywords:** embodied cognition, hand-altered vision, peripersonal space

## Abstract

The effect of hand proximity on vision and visual attention has been well documented. In this study we tested whether such effect(s) would also be present in the auditory modality. With hands placed either near or away from the audio sources, participants performed an auditory-spatial discrimination (Experiment 1: left or right side), pitch discrimination (Experiment 2: high, med, or low tone), and spatial-plus-pitch (Experiment 3: left or right; high, med, or low) discrimination task. In Experiment 1, when hands were away from the audio source, participants consistently responded faster with their right hand regardless of stimulus location. This right hand advantage, however, disappeared in the hands-near condition because of a significant improvement in left hand's reaction time (RT). No effect of hand proximity was found in Experiments 2 or 3, where a choice RT task requiring pitch discrimination was used. Together, these results that the perceptual and attentional effect of hand proximity is not limited to one specific modality, but applicable to the entire “space” near the hands, including stimuli of different modality (at least visual and auditory) within that space. While these findings provide evidence from auditory attention that supports the multimodal account originally raised by Reed et al. (2006), we also discuss the possibility of a dual mechanism hypothesis to reconcile findings from the multimodal and magno/parvocellular account.

## Introduction

The effect of nearby-hands on vision has been well documented since the seminal study by Reed et al. (2006). In a series of studies, Reed et al. found that the placement of a single hand near a potential target location can speed up participants' reaction time (RT) toward that location in the Posner's paradigm (Posner, 1980; Reed et al., 2006). This effect was strongest when one's own hands were visible beside the display, but still remained effective when only visual (i.e., fake hands) or proprioceptive (i.e., covered hands) signals were present (Reed et al., 2006). A series of follow-up experiments by Abrams et al. (2008) found a slower visual search rate when hands were placed near the display, and the authors suggested that the hands perhaps created a stronger but nonselective attentional engagement toward the stimuli around them. This hypothesis would reconcile the seemingly contradictory findings of faster target detection (Reed et al., 2006) and slower visual search (Abrams et al., 2008), because attention is unnecessarily allocated to the distractors in the latter case. Consistent with Abrams et al.'s proposal, subsequent studies also reported slower learning of visual context (Davoli et al., 2012b), slower shift between global and local attention (Davoli et al., 2012a,b,c), and increased accuracy in visual memory tasks (Tseng and Bridgeman, 2011).

To offer a mechanistic explanation for these interesting effects of nearby-hands on visual attention, Reed et al. (2006) suggested the possibility of involvement of multimodal neurons that are located in the frontoparietal network, including the premotor and parietal cortex. These regions have been shown to code objects using a body-centered coordinate system, forming a representation of one's peripersonal space using visual, proprioceptive, tactile, and vestibular information (e.g., Graziano and Botvinick, 2002). The network also selectively responds to both visual and tactile events near the hands (Graziano and Gross, 1995), which accounts for Reed et al.'s (2006) behavioral findings well (for a review, see Tseng et al., 2012; Brockmole et al., 2013). Importantly, studies have now shown that nearby sounds, or auditory information in general, can also elicit responses from these multimodal neurons both in the premotor (Graziano et al., 1999) and parietal cortex (Schlack, 2005), suggesting that auditory information is also integrated into a coherent multimodal or supramodal representation of peripersonal space (Andersen, 1997; Andersen et al., 1997; Serino et al., 2007). One relevant behavioral demonstration comes from Serino et al. (2007), who showed that participants responded to tactile stimulation on the finger faster if a nearby sound was presented (as opposed to a far sound). Although this study is not quite a demonstration of the nearby-hand effect because it is actually the sound that modulates tactile response and not the other way around (e.g., hand presence modulates vision or audition), Serino et al.'s findings nevertheless confirm the possibility of an audio-tactile integration within the peripersonal space.

In light of these findings, the present study investigates whether the effect of hand proximity that has been repeatedly demonstrated in the visuo-tactile domain can also be observed in audition. That is, if the hypothesis of an involvement of the premotor and parietal multimodal neurons offered by Reed et al. (2006) is correct, one should expect to see comparable effects to also take place using auditory stimuli. Thus, in this study we employed a similar two-hands setup used by previous studies (Abrams et al., 2008; Tseng and Bridgeman, 2011), and manipulated hand locations to be either near or far from the audio source (i.e., loudspeakers). Given that previous studies have shown that hand proximity does not generalize to all cognitive tasks, we implemented three different tasks that involved auditory-spatial discrimination, pitch discrimination, and spatial plus pitch discrimination. For example, Davoli et al. (2010) demonstrated that nearby-hands can actually impair the speed of semantic judgment in reading, presumably because the frontoparietal network is more sensitive to spatial information. Therefore, the three experiments included in this study are designed to include both the spatial (location discrimination) and featural (pitch discrimination) components to test whether the same characteristics from vision is also applicable to audition.

## Experiment 1

This experiment used an auditory binary spatial discrimination, a gross form of auditory localization, task, which is analogous to a visual exogenous-orienting task. Participants simply had to respond whether the tone was coming from the left or right, which is a spatial task that relies on purely spatial features of the stimulus. It is reasonable to expect an effect of hand proximity here because similar facilitation in simple RT has been reported in visual orienting paradigms such as a Posner's task (Reed et al., 2006).

### Methods

#### Participants

Twenty participants (10 male and 10 female; mean age = 22) were recruited from the National Central University. All were right-handed, had normal or corrected-to-normal vision, and were naïve to the purpose of this experiment. All participants gave informed consent prior to the start of the experiment and received monetary payment upon completion of the experiment. The experimental apparatus and procedure was approved by the Institutional Review Board of National Cheng Kung University Hospital, Tainan, Taiwan.

#### Apparatus and procedure

**Figure 1:**
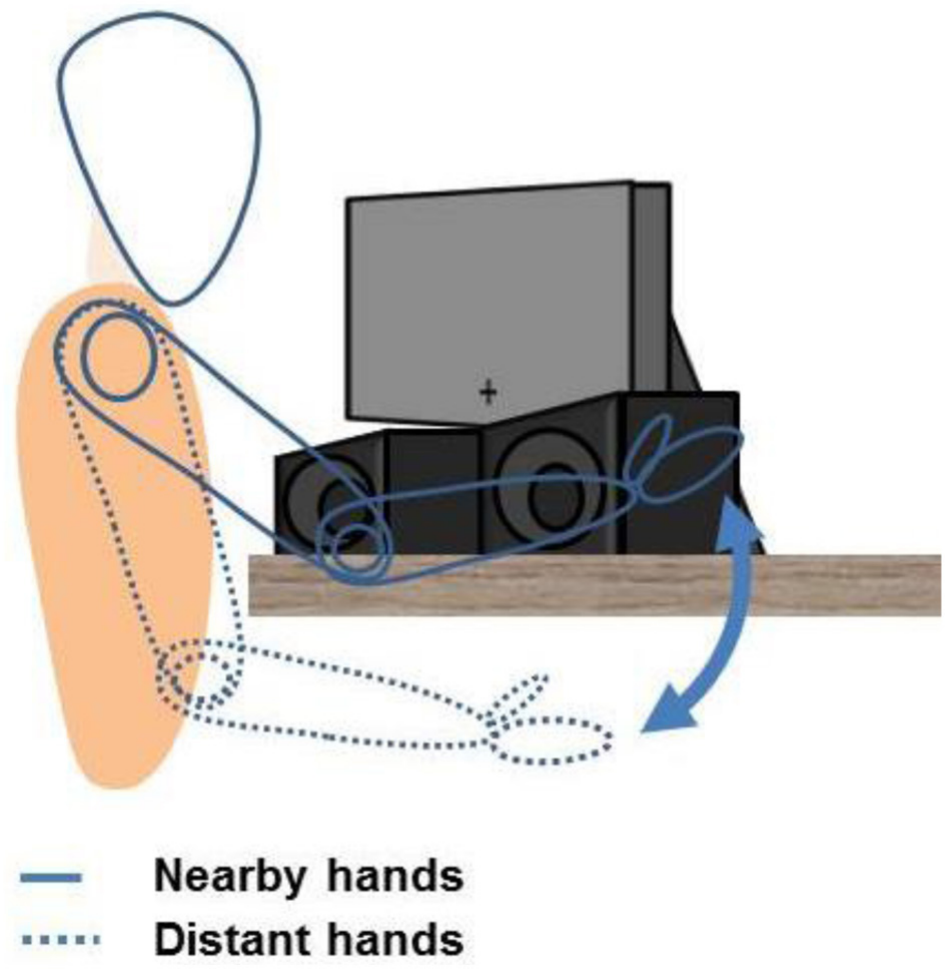
**Apparatus and setup of all experiments**. Participants placed their hands either on their lap or on the table by the speakers, with the distance between hands (approx. 40 cm) fixed by a platform (not shown). Hands are placed vertically to mimic a power grasp. A fixation cross is displayed at the onset of each trial, allowing both speakers (and hands, in the hands-near condition) to remain visible.

The experimental setup consisted of a 15-inch computer display, two speakers, and two response pads. The computer display was positioned approximately 45 cm in front of the participants, and displayed only the fixation cross (at the center and near the bottom of the screen where it is closer to the speakers) at the onset of each trial. The loudspeakers, both left and right, were placed underneath and slightly in front of the display (see Figure 1), approximately 40 cm in front of the participants.

Participants rested their hands on a platform (not shown in Figure 1), where the response pads were mounted. This platform was used for both the hands-near (placed on the table) and hands-far condition (placed on the lap) in order to keep the same gesture and distance intact between both conditions, and also avoid fatigue when the hands were placed on the lap (in the hands-far condition). On this platform, the left and right response pads were approximately 40 cm apart, and both were mounted vertically so that participants' hands mimicked a power grasp position (i.e., left response pad faced left, right response pad faced right, and both palms faced inward with fingers touching the response pads), instead of a flat typing position (see Schultheis and Carlson, 2013; Thomas, 2013, for more on gestures). Throughout the entire experiment, participants placed their hands beside the response pads, with their fingers resting on the response buttons. This gesture was maintained (either on the table or on their lap) throughout the entire block, although participants only had to press the button for a brief period of time for each trial. As such, no arm or elbow movements were required from the participants because the gesture was static, and the participants only had to press the button with their already-in-position fingers.

In the hands-near condition, the left and right speakers were placed within the space between the left and right response pads (Figure 1). The left speaker was aligned against the left response pad, and the right speaker against the right response pad. This arrangement in the hands-near condition was designed to induce the percept for participants were leaning their palms and pressing buttons directly against the sides of the speakers. In the hands-far condition, the platform was moved to the participants' lap but the speakers stayed on the table, and thus everything was kept the same as the hands-near condition except the actual location and visibility of the hands.

On each response pad, there were three buttons (top, middle, bottom), and the participants were instructed to place their index, middle, and ring fingers on the top, middle, and bottom buttons, respectively. Regardless of the different button- and task-requirements between the experiments, this finger-button mapping was used for all the experiments in the present study for the sake of consistency. For all the conditions in this study, participants were instructed to place their chins on a chinrest to avoid any unwanted head movement that would cause unintended perception of uneven volume change coming from the left and right. This also ensured that the ear-to-stimulus distance was kept the same between the hands-near and hands-far conditions.

In Experiment 1, a 600 Hz tone was used. The tone would come from either the left or the right speaker, and participants had to respond with their left or right index fingers by pressing the top button on the left or right response pads, which were placed either by their respective speakers (hands-near condition) or on the participants' left or right lap (hands-far condition). The experiment consisted of two blocks, one hands-near and one hands-far. The order of the blocks was counter-balanced among the participants. In each block, there were 10 practice trials and 60 formal trials. Each trial began with a 1000-ms fixation cross that was positioned toward the bottom of the display, centered above the midline between the two speakers, so that both speakers stayed in view while participants performed the task. This means that although the location of the hands was the key variable of interest here, the visual information associated with the hands inevitably varied between the two conditions (i.e., the hands were not visible to the participants in the hands-far condition). This, however, was done intentionally to maximize the nearby-hand effect because the effect has been suggested to be strongest when one's hands were visible (Reed et al., 2006). The fixation cross was then followed by a 200-ms tone, and participants were told to respond as fast as they could to indicate the side from which the tone came.

### Results and discussion

**Figure 2:**
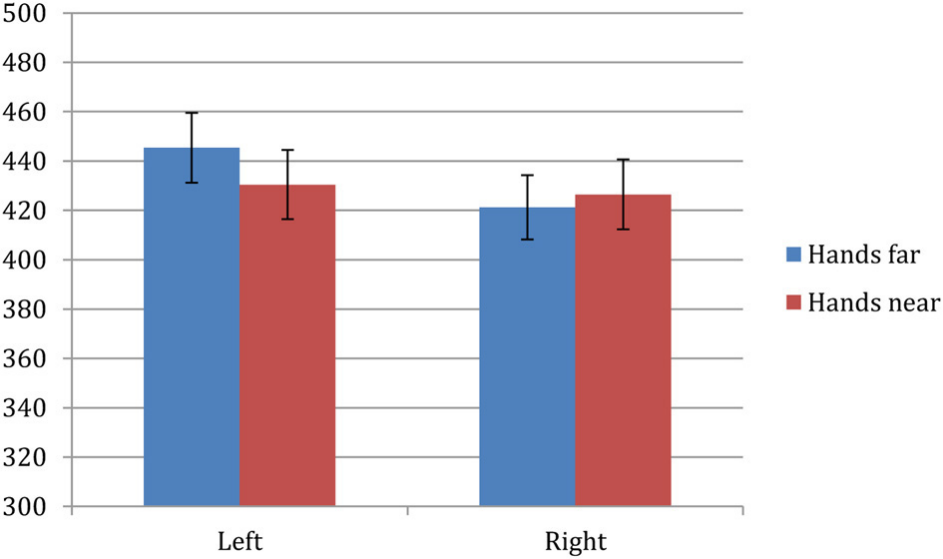
**Experiment 1 results**. ANOVA revealed a significant interaction between hand proximity and laterality (*F* = 8.728, *p* = 0.008), which was driven by a significant difference between the left and right hand in the hands-far condition (right: 421 ms, left: 445 ms, *p* = 0.002) that became nonsignificant in the hands-near condition (right: 426 ms, left: 430 ms, *p* = 0.46). Therefore, left-hand RT was speeded up to be on par with the right hand when one's own hands were near the audio source.

Trials with incorrect responses (<1%) were excluded from data analysis. Remaining data were analyzed with a 2 × 2 repeated-measures ANOVA consisting of factors of hand proximity (near, far) and laterality (left, right). Note that the factor of laterality is simply referring to the tone direction and the responding hand (left or right hand), as the present study used a two-hands setup in the hands-near condition that is similar to Abrams et al. (2008) and Tseng and Bridgeman (2011) instead of the single hand setup by Reed et al. (2006). The effect of hand proximity did not reach statistical significance (*F* = 0.106, *p* = 0.748), but there was a significant effect of laterality (*F* = 8.355, *p* = 0.009) and significant interaction between hand proximity and laterality (*F* = 8.728, *p* = 0.008). *Post-hoc* comparisons for the main effect between left and right hand showed a significantly faster RT for the right hand (423.84 ms) over the left hand (437.91 ms). However, *post-hoc* comparisons for the interaction further clarified that the right hand was only faster than the left in the hands-far condition (right: 421 ms, left: 445 ms, *p* = 0.002), whereas the left hand was equally fast as the right hand in the hands-near condition (right: 426 ms, left: 430 ms, *p* = 0.46). Therefore, left-hand RT decreased to be on par with that of the right hand when one's hands were near the audio source (Figure 2).

From the results summarized above, we found that auditory localization was facilitated in the left hand when one's hands were both near the auditory source. Specifically, this lack of difference between the left and right hand in the nearby-hand condition was due to the left hand speeding up, relative to the left hand-far condition. The binary auditory localization task used here is somewhat similar to the auditory analogue of a visual detection task, which has been used previously to assess the timing cost of inter-hemispheric transmission (e.g., Jeeves, 1969, 1972; Berlucchi et al., 1971). In the visual detection task, a flash of light is presented either to the left or right visual field. Responses made with the ipsilateral (to the stimulus) hand are slightly but significantly faster than the contralateral hand. The same trend persists even when both hands are crossed, suggesting that the RT difference is best explained by an anatomical account instead of spatial compatibility (Berlucchi et al., 1977). In theory, this is because the perception of the stimulus, as well as the control of the ipsilateral hand, are both mediated by the contralateral hemisphere; whereas the ipsilateral hemisphere would require additional traveling of the signals through the corpus collosum to the other hemisphere for motor output. Unlike these studies, however, in this experiment we did not manipulate the ipsi- and contralateral aspects of the responding hand. That is, in this experiment the left and right hand was always assigned to respond left and right, respectively, thereby maintaining the optimal ipsilateral RT as described by previous studies. Yet, we still observed a right hand advantage over the left hand in RT in the control (hands-far) condition. Indeed, in addition to the ipsilateral hand advantage in RT, when all things are held equal, studies have found that right-handers are consistently faster when responding with their right hand. This is true in detecting visual events (Berlucchi et al., 1971) and using a computer mouse (Peters and Ivanoff, 1999), and has been attributed to the left-hemisphere dominance in right-handers (Berlucchi et al., 1971, 1977). Following this logic, we speculate that hand proximity may have enhanced left-hand RT by bringing participants' right-hemisphere activation above a certain threshold. But, perhaps a more intuitive explanation is that most right-handers have simply hit a ceiling level of response speed with their over-rehearsed right hand. Nevertheless, the left-hand advantage observed here corresponds well with Reed et al.'s (2006) original report of a left hand/side RT advantage, and the current demonstration of the effect of hand proximity in auditory processing supports their multimodal neuron account.

## Experiment 2

In Experiment 1 we observed an effect of hand proximity on binary location discrimination on the left side. However, whether this advantage can be transferred to other forms of auditory tasks, such as featural discrimination, remains to be investigated. One interesting aspect of most nearby-hand studies to date is the spatial nature of many “facilitated” tasks: visual memory (color-location binding), visual search, shifting of visual attention. Therefore, it would be helpful to know if hand proximity would facilitate other processes in the auditory modality when such spatial information is either degraded or made less salient. To this end, in Experiment 2 we used a unidirectional pitch discrimination task to test whether nearby hands would facilitate auditory processing beyond simple discrimination tasks.

### Methods

#### Participants

A new group of 20 participants (10 male and 10 female; mean age = 21) that did not participate in Experiment 1 were recruited from the National Central University. All were right-handed, had normal or corrected-to-normal vision, and were naïve to the purpose of this experiment. All participants gave informed consent prior to the start of the experiment and received monetary payment upon completion of the experiment. The experimental apparatus and procedure was approved by the Institutional Review Board of National Cheng Kung University Hospital, Tainan, Taiwan. All participants performed well during the practice block and thus no one was excluded from further analysis.

#### Apparatus and procedure

The apparatus and procedure were mostly identical to those of Experiment 1, with the following exceptions. First, three tones at 400, 600, and 800 Hz were used in the current experiment. For simplicity's sake, we label them as low (400 Hz), med (600 Hz), and high (800 Hz) in this report. Second, the tones emanated from both speakers, thus there was no left or right judgment for this task. Third, participants still placed both of their hands by the speakers, but were instructed to only respond with their dominant (right) hand, using their index, middle, and ring fingers, to indicate high, med, and low tones, respectively. Since the hands were vertically positioned like in Experiment 1 in order to mimic a power grasp (Thomas, 2013), the right fingers were naturally positioned with the index finger at the higher position, the middle finger in the middle, and the ring finger at the lower position. Therefore, participants were told that their high (index), med (middle), and low (ring) positioned fingers are designed to correspond to the high, med, and low tones, respectively, to avoid any confusion over stimulus-response compatibility. Both the hands-near and hands-far blocks began with 21 practice trials, followed by 60 formal trials.

### Results and discussion

Trials with incorrect responses (<4%) were excluded from data analysis. Remaining data were submitted to a 2 × 2 repeated-measures ANOVA consisting of factors of hand proximity (near, far) and pitch (high, med, low). There was a main effect of pitch (*F* = 6.712, *p* = 0.003), but no significant effect for hand proximity (*F* = 0.009, *p* = 0.926) or interaction between hand proximity and pitch (*F* = 0.215, *p* = 0.808). *Post-hoc* comparisons between high, med, and low tone RTs showed that participants responded faster toward high tones (577 ms) than low (*p* = 0.012) and med (*p* = 0.002) tones, whereas low (633 ms) and med (643 ms) tone RTs were not different from each other (*p* = 0.614). Therefore, there was no effect of hand proximity in this pitch discrimination task.

**Figure 3:**
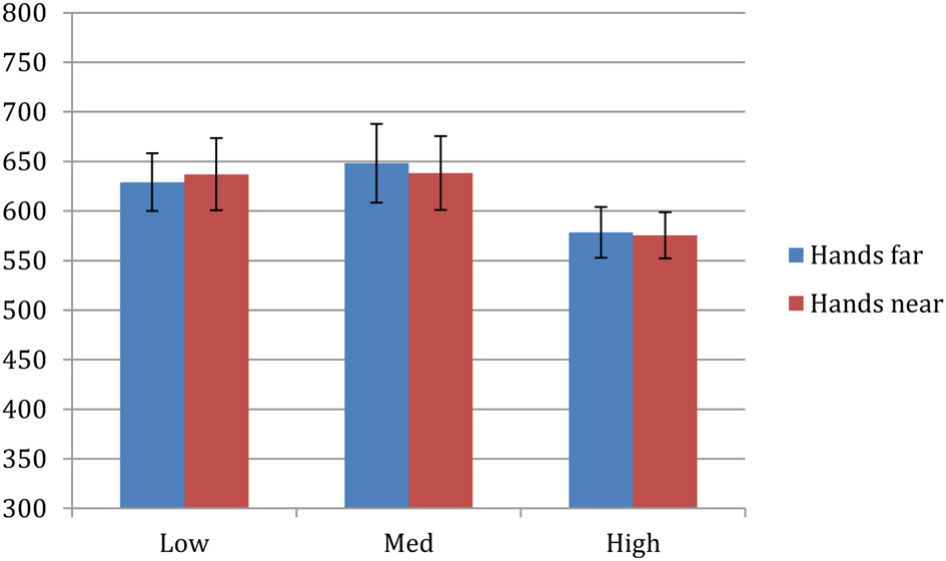
**Experiment 2 results**. There was a main effect of pitch (*F* = 6.712, *p* = 0.003), but no significant effect for hand proximity (*F* = 0.009, *p* = 0.926) or interaction between hand proximity and pitch (*F* = 0.215, *p* = 0.808).

In this experiment we observed faster RT toward the high tone, presumably because the index finger was faster in pressing buttons than the middle and ring fingers. However, there was no effect of hand proximity in any of the three tones (Figure 3). This null result is somewhat surprising, but is consistent with the idea that the effect of hand proximity seem to be less robust when the spatial component in the task is less salient. It is also worth noting that, unlike previous findings, we did not observe a nearby-hand impairment effect here either. This useful difference suggests that the enhanced visual-analysis account that is responsible for the slower shifts of visual attention (Abrams et al., 2008; Tseng et al., 2012; Brockmole et al., 2013) is not applicable to auditory processing; otherwise we should observe a slower RT in the hands-near condition because too much attention is unnecessarily devoted to auditory discrimination. Therefore, auditory processing seems to be less sensitive to the effect of hand proximity.

There are two possible explanations that can account for the findings from Experiments 1 and 2 so far. In Experiment 1, the speeded location discrimination task is essentially a simple RT task, whereas the discrimination task here is a choice RT task due to an additional stage of detailed, featural differentiation. Thus, the first possibility is that nearby-hands are only weakly effective in modulating auditory attention or auditory peripersonal space, and that such an effect is present in simple RT tasks but becomes *insufficient* when more cognitive resources are demanded, such as the case in Experiment 2. The second possibility is that perhaps nearby-hands are sufficient enough to improve RT in both experiments, but did not in Experiment 2 due to *inapplicable* task demands. The rationale for this possibility comes from what we speculate might be a key difference between the current auditory discrimination task and the rest of the literature on visuo-tactile facilitation—the degree of involvement of spatial attention. That is, most visuo-tactile experiments in the current literature are highly spatially-oriented: visual working memory requires featural-spatial binding (Tseng and Bridgeman, 2011) and visual search requires spatial shifts of attention (Abrams et al., 2008). The same applies to visuo-spatial learning (Davoli et al., 2012b) and shifting between global and local scopes of attention (Davoli et al., 2012a,b,c), as well as auditory location discrimination in Experiment 1. Therefore, it is possible that hand proximity does not modulate all aspects of cognitive performance, but only those that would benefit from enhanced spatial attention, be it in vision or audition. In the context of the auditory discrimination task here, then, although spatial attention was indeed enhanced by hand proximity, the unidirectional nature of these acoustic pitches (that are not situated differently in space) could not benefit from an enhanced spatial attention. To test between these two possibilities, a new experiment is needed by adding the localization component to the current discrimination task, so that the discrimination task now becomes spatially relevant.

## Experiment 3

The results from Experiments 1 and 2 raise two possibilities. First, hand proximity may simply be insufficient to modulate anything beyond simple binary localization in the auditory modality. An alternative explanation is that a non-spatial auditory discrimination task is not spatially salient enough to uncover the effect of hand proximity. To test between these two accounts, here we combined the speeded binary localization (Experiment 1) and pitch discrimination (Experiment 2) tasks into one task such that auditory discrimination is now spatially relevant. That is, the high, med, and low tones will either come from the left or right speakers, thus participants must direct their attention spatially and perform the discrimination task. With this design, the “*insufficient*” hypothesis would predict a null result because although even with a spatial task, the task still involves choice RT and is therefore too complex or beyond the involvement of nearby-hand mechanism in auditory peripersonal space. Alternatively, the “*inapplicable*” hypothesis would predict that, with the task being highly spatial (different pitch situated in two different locations), participants would benefit from enhanced spatial attention and show enhanced localization and discrimination.

### Methods

#### Participants

A new group of 20 participants (10 male and 10 female; mean age = 22) that did not participate in Experiments 1 and 2 were recruited from the National Central University. All were right-handed, had normal or corrected-to-normal vision, and were naïve to the purpose of this experiment. All participants gave informed consent prior at the start of the experiment and received monetary payment upon completion of the experiment. The experimental apparatus and procedure was approved by the Institutional Review Board of National Cheng Kung University Hospital, Tainan, Taiwan.

#### Apparatus and procedure

The task here was a combination of the localization and discrimination task from Experiments 1 and 2. Participants positioned both hands vertically in a power grasp gesture, and placed them either by the speakers (hands-near) or on their lap (hands-far). The vertical hand placement allowed the same consistent stimulus-response mapping from Experiment 2, where the index finger is positioned on top and is associated with the high tone, the middle finger is positioned in the middle and is associated with the med tone, and the ring finger is positioned at the bottom and is associated with the low tone. The tones were the same as those used in Experiment 2. Critically, the tones would either come from the left or the right speaker like the localization task in Experiment 1, and participants needed to use the correct hand (left, right) and the correct finger (high, med, low) to respond. For example, a med tone from the left should be responded by the left middle finger, and a high tone from the right should be responded by the right index finger. In each block (hands-near, hands-far), participants performed 30 practice trials and 180 formal trials. Everything else was the same as Experiments 1 and 2.

### Results and discussion

Trials with incorrect responses (<5%) were excluded from data analysis. Remaining data were analyzed with a 2 × 2 × 3 repeated-measures ANOVA consisting factors of hand proximity (hands-far, hands-near), laterality (left, right), and pitch (high, med, low). There was a significant main effect of laterality (*F* = 10.134, *p* = 0.005) and pitch (*F* = 4.201, *p* = 0.022), but not hand proximity (*F* = 1.357, *p* = 0.258). None of the interaction terms were statistically significant. Separate comparisons under laterality revealed that, like Experiment 1, participants' right hand responses were significantly faster than their left hand responses (*p* = 0.005). Separate comparisons under pitch also revealed that, like Experiment 2, participants' responses toward the high tone were significantly faster than those toward the low (*p* = 0.051) and med tones (*p* = 0.018). Finally, and most importantly, we did not observe the critical finding from Experiment 1, namely faster RT for the left hand in auditory localization when hands are within close proximity of the auditory stimuli.

**Figure 4:**
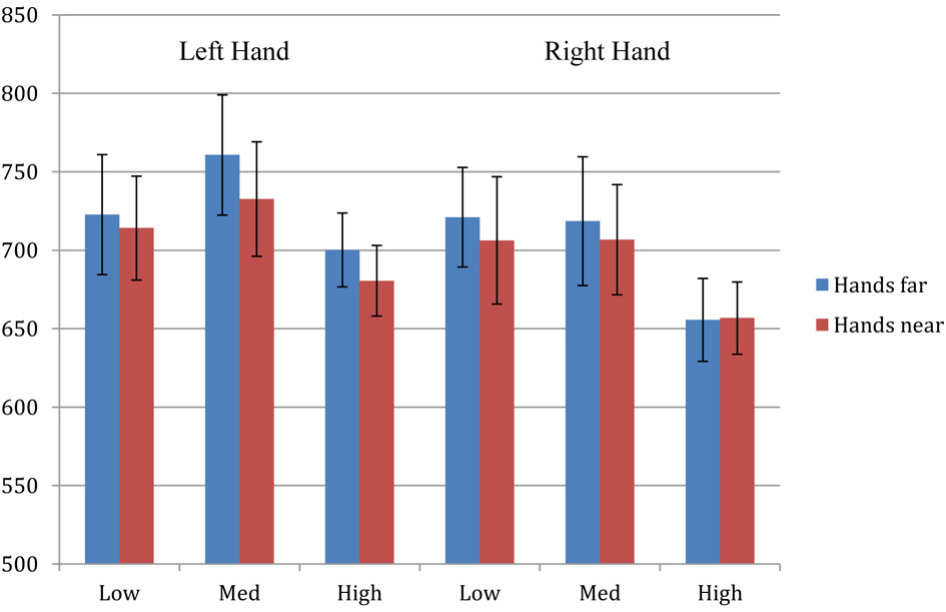
**Experiment 3 results**. ANOVA revealed a significant main effect of laterality (*F* = 10.134, *p* = 0.005) and pitch (*F* = 4.201, *p* = 0.022), but not hand proximity (*F* = 1.357, *p* = 0.258) or other interaction terms. Separate comparisons under laterality revealed that, like Experiment 1, participants' right hand responses were significantly faster than their left hand responses (*p* = 0.005). Separate comparisons under pitch also revealed that, like Experiment 2, participants' responses toward the high tone were significantly faster than those toward the low (*p* = 0.051) and med tones (*p* = 0.018). Thus, all the general effects from Experiments 1 and 2 are replicated, except the effect of hand proximity.

The absence of the nearby-hand effect suggests that the effect is much weaker in the auditory domain, and cannot support auditory processing beyond simple RT tasks. While one could argue that the current task might have been too difficult, or was still not spatial enough to rule out the second hypothesis, we think this is unlikely for several reasons: (1) the localization and discrimination protocol here is identical to those from the first two experiments, (2) we did replicate the general right-hand RT advantage from Experiment 1, suggesting that the current task indeed contained the critical spatial component that was necessary, and (3) we also replicated the high-pitch index-finger advantage from Experiment 2, thus every general effect was replicated except the specific effect of hand proximity. Taken together, we have successfully replicated the right-hand and index-finger RT advantage from Experiments 1 and 2, respectively. These replications of the motor-related effects suggest that the motor programming codes between all three experiments are quite consistent, and therefore the lack of replication of the hand proximity effect is less likely to be attributable to the more complex format of motor response in Experiment 3. Although there is a slightly bigger RT decrease in the left hand when hands are nearby, the magnitude does not reach statistical significance (Figure 4). Together, these results support the *insufficient* hypothesis from the previous two experiments, and suggest that hand proximity has a significant but limited effect in altering auditory processing. Consequently, when performing a simpler spatial discrimination task, the (left) hand proximity effect is observed, but when performing a more complex discrimination task, this proximity effect is eliminated.

## General discussion

The present study was set out to test whether multimodal neurons are likely the neural mechanism underlying the effect of hand proximity in vision (e.g., Reed et al., 2006; Tseng et al., 2012; Brockmole et al., 2013). To this end, we utilized the auditory characteristics of these neurons and tested whether the effect of hand proximity in vision can also be observed in audition. In Experiment 1, we found that binary spatial discrimination became faster for the left hand/side when both hands are near the audio source, providing support for Reed et al.'s (2006) original report of left hand/side RT advantage, as well as their multimodal neuron account for the facilitatory effect. In Experiment 2, using a non-spatial pitch discrimination task that is measured by choice RT, we found no effect of hand proximity, neither facilitation nor impairment. This could be due to the fact that the spatial aspect—an important component for the effect of hand proximity—was taken out with the unidirectional audio setup, or the fact that choice RT was just too complex for the hand effect, at least in the auditory modality (but certainly not in vision). These competing explanations were resolved in Experiment 3, where we reintroduced the binary spatial discrimination element from Experiment 1, together with pitch discrimination, in order to make the pitch choice RT task more spatially relevant: we again found no effect of hand proximity, suggesting that the complex choice-response task was the key to why the hand effect failed to facilitate performance. Together, these results suggest that (1) the effect of hand proximity is not exclusive to vision, but can also enhance auditory processing to certain extent, and (2) the multimodal neuron hypothesis originally provided by Reed et al. (2006) is supported by the current findings, and (3) the effect of hand proximity is weaker in audition than in vision since only auditory-spatial discrimination, but not tone discrimination, is enhanced.

It is important to note that the present findings cannot be explained by stimulus-response compatibility (Simon, 1968; Simon et al., 1970; Lloyd et al., 2010) or the comfort level of the hands. First, in the present study, the left hand was always responding to the left stimulus and the right hand was always responding to the right stimulus (Experiments 1 and 3). The upper fingers always responded to the high tone, the middle finger to the med tone, and the lower finger to the low tone (Experiments 2 and 3). These stimulus-compatible patterns were the only ones used, and stayed the same throughout the entire experiment; thus there were no response configurations that were incompatible with the stimulus. Second, regarding hand comfort, one notable study has already demonstrated that the effect of hand proximity in vision cannot be attributed to the posture or comfort that is associated with the nearby-hand setup (Weidler and Abrams, 2013). But most importantly, if the effect we have observed here was purely driven by easier positioning of the hands, then we should have observed faster RT for the right hand in Experiment 1 and for both hands in Experiment 2, but this was not the case and the facilitatory effect of hand proximity was not only left-hand specific, but also task specific.

### Right hand advantage in the hands-away condition

The critical finding from the present study is the improved RT in the left hand when performing auditory binary spatial discrimination (a gross form of localization). A closer examination of Figure 2 suggests that the right hand was initially faster than the left (hands-far condition), but that difference was no longer present in the hands-near condition due to faster performance in the left hand, rather than slower performance in the right (Experiments 1 and 3). It is curious why, in the default setting (hands away), there may be left vs. right hand asymmetry in RT in the first place. In vision, it has been repeatedly shown that right-handers respond faster with their right hand even when the two hands are crossed (Berlucchi et al., 1971). This is likely because the right hand is very well-rehearsed in all tasks (at least in right-handers), and therefore it is always operating at the ceiling level; such ceiling performance would explain why the right hand could not benefit from hand proximity. Another possibility, though less intuitive, is that perhaps most right-handed individuals are mostly left-hemisphere dominant (Berlucchi et al., 1977), and therefore would respond quicker with their contralateral right hand and slower with their ipsilateral left hand (due to inter-hemispheric transmission). Further research has suggested that although this anatomical lag persists in all kinds of tasks, the magnitude of the lag is clearest in simple RT paradigms, and becomes less clear or even nonexistent in choice RT paradigms due to the additional processes that are involved (e.g., Anzola et al., 1977), accurately reflecting what was observed in the present study. As such, it is also reasonable that our participants (all right-handed) would show a right-hand advantage in tasks requiring auditory spatial discrimination.

### Theoretical implications

In the context of a speeded RT paradigm, we think the left-hand improvement possibly implies an increasingly active right hemisphere induced by the hands (see Langerak et al., 2013, for a recent example). A similar idea has been proposed before to explain the effect of hand proximity in vision (Bridgeman and Tseng, 2011; Tseng et al., 2012), because incidentally the right parietal cortex (where the multimodal neurons are) is heavily involved in the process of multisensory integration, and would also provide a consistent account for the current findings. More direct evidence for a right hemisphere involvement comes from a neuroimaging study by Brozzoli et al. (2012). These authors used fMRI to investigate the remapping of hand-centered space while their participants experienced the rubber hand illusion. They found that functionally, the degree of remapping of hand-centered space was strongly correlated with activities in the right posterior parietal cortex, while phenomenologically, the degree of conscious feeling of ownership over the fake hand is correlated with activities in the left premotor cortex. This dissociation between the roles of the right parietal cortex and left premotor cortex is quite informative, and implies that the effect of hand proximity likely recruits the parietal cortex. This explanation is also consistent with all previous reports of the effect of hand proximity in vision, because the right parietal lobe is not only involved in multisensory integration, it is also associated with a multimodal, or supramodal, representation of space and spatial attention (e.g., Farah et al., 1989; Molholm, 2006; Rushworth and Taylor, 2006). Note that, however, here we assume the effect of hand proximity in vision and audition is mediated by a common set of multisensory neurons, or at least different multisensory neurons located within the same brain region. It remains possible that this may not be the case, and an alternative possibility is that visual and auditory modalities exhibit similar laterality effects and are therefore likely to be similarly lateralized in the brain.

Besides the multimodal neuron hypothesis raised by Reed et al. (2006), recently a new hypothesis that suggests the magnocellular pathway as a possible mechanism for the nearby-hand effect has also received much empirical support (Gozli et al., 2012; Abrams and Weidler, 2013; Chan et al., 2013). The parvo/magno hypothesis states that nearby-hands automatically biases the visual system to recruit the magnocellular pathway more, which processes visual information rapidly while sacrificing details such as colors. However, it is unclear how such mechanism in vision can account for the current findings here in audition. In addition, one advantage of the multimodal account is that it is not limited to the stage of perception. That is, although nearby-hands can modulate visual processing early at the perception level (e.g., Brown et al., 2008; Cosman and Vecera, 2010; Gozli et al., 2012), it can also have later effects at the attention level such as semantic judgment (Davoli et al., 2010), attentional shielding (Davoli and Brockmole, 2012), tool functionality processing (Reed et al., 2010), or joint-attention processing (Sun and Thomas, 2013). Perhaps a third alternative is that there may possibly be dual mechanisms for the nearby-hand effect in vision (multisensory plus parvo/magno), but not in audition (multisensory only, without parvo/magno). This idea would explain why the observed effect of hand proximity here is much weaker in audition than vision. Further research is necessary to test whether this dual-mechanism account of hand proximity is feasible or not. Nevertheless, the most important theoretical contribution of the current findings would be the demonstration of the effect of hand proximity on auditory stimuli. This implies that the perceptual and attentional effect of hand proximity is not limited to one specific modality, but applicable to the entire “space” near the hands, including whatever stimuli (at least visual and auditory) within that space. This would also be consistent with the abovementioned supramodal representation of space (Farah et al., 1989).

### An alternative explanation for the left hand improvement

Although we favor the right parietal cortex as responsible for the left hand improvement in auditory localization (Tseng et al., 2012), an alternative explanation should also be considered. Specifically, it remains possible that there was no effect of hand proximity in the right hand because the right hand treats both near and far distances as within the peripersonal space. In other words, the extent of the peripersonal space is asymmetrical between the left and right hand, with the right hand enjoying an augmented peripersonal space (Peters and Ivanoff, 1999). Support for this idea comes from studies showing that tool-use can temporarily but effectively augment auditory peripersonal space (Serino et al., 2007), and the right hand's extended training in using computer mouse and keypad makes it possible to shrink the far space into near (Bassolino et al., 2010) because the right hand is used to acting on these near-hand devices while observing the effects of such actions take place in far space (i.e., on the computer screen far away). From this perspective, the effect of hand proximity is actually present in the right hand, both the hands-near and hands-far conditions (Experiment 1) because the far space is effectively treated as near. However, there is one point in our study that goes against this explanation. The original Bassolino et al. study (2010) reported a shrinking far-space in right hands due to mouse usage. This effect is unlikely to transfer to keyboard or keypads in the current study because the left hand, although less adept in using a mouse, is completely adept in using a keyboard since typing requires both hands regardless of one's handedness. Therefore, one would expect a null finding in the left hand in Experiment 1 with equally fast RT as the right hand in both hands-near and hands-far conditions, if extended training on keypad was indeed effective in shrinking the far space.

### Inconsistent left- and right-hand advantage in the effect of hand proximity

The current finding of a left hand/side RT improvement is consistent with the finding of Reed et al. (2006), but presents a sharp contrast with the right hand/side advantage reported by Tseng and Bridgeman (2011). Indeed, the kinds of tasks that are used by different studies seem to show different sides of attentional prioritization and bias (for a review, see Tseng et al., 2012). Previously, Tseng and Bridgeman (2011) proposed a functional account that aims to explain the right hand bias as a reflection of the frequency of the uses of each hand (also see Reed et al., 2010, for a similar account in tool-use). In light of our current finding on a left hand improvement, our working hypothesis is that perhaps a simpler detection type of task, such as the speeded localization task from Experiment 1 here and the Posner's paradigm (i.e., detecting visual targets) employed by Reed et al. (2006), especially measured in RT, can be shown in the form of a left hand advantage. The right hand advantage, on the other hand, is likely a result of the top-down influence (e.g., Garza et al., 2013) that prompts observers to attend right (the functional account), as shown by the more complex visual discrimination task (Lloyd et al., 2010) and change detection task (Tseng and Bridgeman, 2011) that requires accuracy and not speed. This hypothesis will need further testing and fine-tuning, and will certainly need to include the interaction between task type and one's handedness, as it has been shown that one's handedness can also change the area of attentional prioritization (Le Bigot and Grosjean, 2012). As previously mentioned, a difference between left- and right-hand ceiling performances may also contribute to whether the effect of hand proximity is observable. Future research is needed to determine whether individual differences in such laterality of the effect is a result of anatomical hemisphere dominance, differential ceiling between the left- and the right-hand, top-down attentional bias that is learned over time (Reed et al., 2010; Tseng and Bridgeman, 2011), or all of the above.

## Conclusion

In this study, we demonstrate that the effect of hand proximity that is often observed in vision can also be observed in audition when hands are placed near the audio source. Interestingly, this effect is only present in an auditory location discrimination task (simple RT; Experiment 1), and disappears when complex judgment such as pitch discrimination is required (choice RT; Experiments 2 and 3). Furthermore, the facilitative effect only exists in left hand RT. We take these results as evidence supporting Reed et al.'s original multisensory account (2006). We also note that the effect in audition is perhaps weaker than vision, which leaves open the possibility of a dual-mechanism account (multisensory plus magno/parvo) that is exclusive to vision but not audition. The current finding also raises new questions regarding the effect of hand proximity, such as the role of hemispheric difference and top-down attentional bias in initiating the effect, and whether there is a systematic pattern underlying the laterality of the nearby-hand effect, all of which remains to be addressed by future studies.

### Conflict of interest statement

The authors declare that the research was conducted in the absence of any commercial or financial relationships that could be construed as a potential conflict of interest.
